# 

**Published:** 1888-11-17

**Authors:** 


					i PERMANENTLY ENLARGED TO 32 PAGES.
price one penny.
112, Vol. V.-
-November 17th, 1888.
Cttegle
tered at the General Post Office
as a Newspaper.]
Per Annum, 6s. 6d, post free.
Monthly Parts. 6d.; post fIee> 7id. D
Ditto per Ann., 7s. 6d. post free. U
A Weekly Institutional Journal of
Srietttt, /Iftciitchtt, IFUtrsing, attir l^ljilautijrDpg

				

